# Menthol Pretreatment Alleviates *Campylobacter jejuni*-Induced Enterocolitis in Human Gut Microbiota-Associated IL-10^−/−^ Mice

**DOI:** 10.3390/biom14030290

**Published:** 2024-02-29

**Authors:** Markus M. Heimesaat, Luis Q. Langfeld, Niklas Schabbel, Nizar W. Shayya, Soraya Mousavi, Stefan Bereswill

**Affiliations:** Gastrointestinal Microbiology Research Group, Institute of Microbiology, Infectious Diseases and Immunology, Charité-Universitätsmedizin Berlin, Corporate Member of Freie Universität Berlin, Humboldt-Universität zu Berlin, and Berlin Institute of Health, 12203 Berlin, Germany

**Keywords:** menthol, immune-modulatory properties, anti-oxidant effects, human gut microbiota-associated IL-10^−/−^ mice, campylobacteriosis model, host-pathogen interaction, *Campylobacter jejuni*, placebo-controlled preclinical intervention study, pretreatment

## Abstract

Human *Campylobacter jejuni* infections are of worldwide importance and represent the most commonly reported bacterial enteritis cases in middle- and high-income countries. Since antibiotics are usually not indicated and the severity of campylobacteriosis is directly linked to the risk of developing post-infectious complications, non-toxic antibiotic-independent treatment approaches are highly desirable. Given its health-promoting properties, including anti-microbial and anti-inflammatory activities, we tested the disease-alleviating effects of oral menthol in murine campylobacteriosis. Therefore, human gut microbiota-associated IL-10^−/−^ mice were orally subjected to synthetic menthol starting a week before *C. jejuni* infection and followed up until day 6 post-infection. Whereas menthol pretreatment did not improve campylobacteriosis symptoms, it resulted in reduced colonic *C. jejuni* numbers and alleviated both macroscopic and microscopic aspects of *C. jejuni* infection in pretreated mice vs. controls. Menthol pretreatment dampened the recruitment of macrophages, monocytes, and T lymphocytes to colonic sites of infection, which was accompanied by mitigated intestinal nitric oxide secretion. Furthermore, menthol pretreatment had only marginal effects on the human fecal gut microbiota composition during the *C. jejuni* infection. In conclusion, the results of this preclinical placebo-controlled intervention study provide evidence that menthol application constitutes a promising way to tackle acute campylobacteriosis, thereby reducing the risk for post-infectious complications.

## 1. Introduction

*Campylobacter* are microaerophilic and rod-shaped Gram-negative bacilli that typically reside as commensal members of the intestinal tract in warm-blooded vertebrates [1]. Frequently colonized with *Campylobacter jejuni*, birds, in particular chickens and turkeys, rarely display clinical signs [2,3,4]. During the livestock slaughter process, the derived meat products might become contaminated with bacteria [5]. Upon human consumption of undercooked or even raw contaminated meat products, unpasteurized milk or surface water, *C. jejuni* infection can occur, leading to acute campylobacteriosis [6,7]. After a latency of 2 to 5 days, infected individual presents symptoms of varying severity, including general malaise, tenesmus, nausea, vomiting, and fever accompanied by enteritis with watery or bloody diarrhea accompanied by mucous discharge [8,9]. The severity of the *C. jejuni*-induced disease depends on both the arsenal of virulence factors of the enteropathogen and the immune competence of infected individuals [10,11]. Following successful passage of the stomach and duodenum, the motile *C. jejuni* traverse the viscous mucous barrier, reach the subepithelial layers by invasion of epithelial cells, and initiate macrophage activation, resulting in the recruitment of monocytes, neutrophilic granulocytes, and leukocytes to the infected sites [12,13]. Furthermore, the endotoxin lipo-oligosacchride (LOS) leads to a Toll-like receptor-4 (TLR-4)-dependent overstimulation of the host immune system [11]. Consequently, pro-inflammatory mediators aim to limit the infection but cause damage to intestinal tissues and induce even systemic complications [11]. The oxidative stress results in gut epithelial cell apoptosis, compromised epithelial barrier function, malabsorption and diarrhea with electrolyte loss (“leaky gut”) [14,15,16]. Typically, the treatment of symptomatic *C. jejuni*-infected individuals involves non-steroidal antiphlogistic compounds and the substitution of fluids and electrolytes [9]. In rather critical cases, particularly in patients with immune-compromising co-morbidities, however, antibiotics such as macrolides or fluoroquinolones may be prescribed [9,17]. Generally, enteritis symptoms completely resolve within two weeks post-infection (p.i.). However, on rare occasions, autoimmune disorders might manifest with a latency of a few weeks to months p.i. [18]. These post-infectious complications of *C. jejuni* infection can affect the joints (reactive arthritis), the intestines (irritable bowel syndrome, inflammatory bowel diseases), or the nervous system (Guillain–Barré syndrome) [19,20,21] and are known to depend on the sialylation status of the *C. jejuni*-LOS [18,22]. Notably, there is a direct correlation between the risk of developing such autoimmune diseases and the severity of the preceding enteritis [22]. In addition, given the rising prevalence of multi-drug-resistant *C. jejuni* strains, which limits effective antibacterial treatment of severely ill patients [17,23], it is important to identify non-toxic compounds with potential anti-*C. jejuni* and anti-inflammatory properties that alleviate the disease and, in turn, reduce the risk of post-infectious diseases. This urgency is even further emphasized by the increasing global incidence of campylobacteriosis cases, which represents the most commonly reported cause of bacterial enteritis, with unreported cases far exceeding the reported ones [24]. In 2021, more than 127,000 campylobacteriosis cases were confirmed in the European Union alone, for instance [25], and the associated expenses for society, including the economies and health care systems, have been shown to be significant [26,27,28].

Menthol, a monoterpene with a high abundance of essential oils originating from the peppermint plant *Mentha piperita L.* (*Lamiaceae*), is widely employed as a flavoring agent and a remedy in traditional folk medicine due to its various health-promoting properties [29]. In fact, menthol has been shown to exhibit potent anti-microbial, anti-inflammatory, anti-oxidant, and anti-apoptotic effects [30,31,32,33,34], positioning the compound as a promising candidate for combating *C. jejuni*-induced morbidities.

Therefore, we tested the potential of prophylactic menthol treatment in experimental campylobacteriosis, focusing on its pathogen-reducing, immunomodulatory and consequent disease-alleviating effects. To address this, we utilized human microbiota-associated (hma) IL-10^−/−^ mice. Given that the commensal gut microbiota of murine (as opposed to human) origin protects against *C. jejuni* infection [35], we initially depleted the murine gut microbiota by antibiotic treatment and subsequently introduced a complex human gut microbiota instead by fecal microbiota transplantation (FMT). Interestingly, mice demonstrate higher resistance to bacterial TLR-4 ligands, including lipo-polysaccharide (LPS) and LOS [36]; however, the *il10* gene deficiency, encoding the anti-inflammatory cytokine IL-10, can overcome this LPS and LOS resistance [37]. In fact, microbiota-depleted wildtype mice were shown not to develop overt *C. jejuni*-induced enteritis [35]. In contrast, within less than a week, both microbiota-depleted and hma IL-10^−/−^ mice succumbed to *C. jejuni*-induced disease affecting not only the intestinal tract but also extra-intestinal and systemic organs [38,39]. Hence, the hma IL-10^−/−^ mouse model serves as a valuable experimental tool to explore the effects of the compound of interest on the interactions among (i) the enteropathogen (i.e., *C. jejuni*), (ii) the human gut microbial communities, and (iii) the vertebrate host immunity during acute campylobacteriosis [39]. This was the major driver behind subjecting hma IL-10^−/−^ mice to peroral menthol treatment starting a week before *C. jejuni* infections to monitor intestinal pathogen burdens, clinical, macroscopic, and microscopic complications of infection, as well as pro-inflammatory immune responses in the intestinal tract and beyond.

## 2. Materials and Methods

### 2.1. Mice, Microbiota Depletion

**Figure 1:**
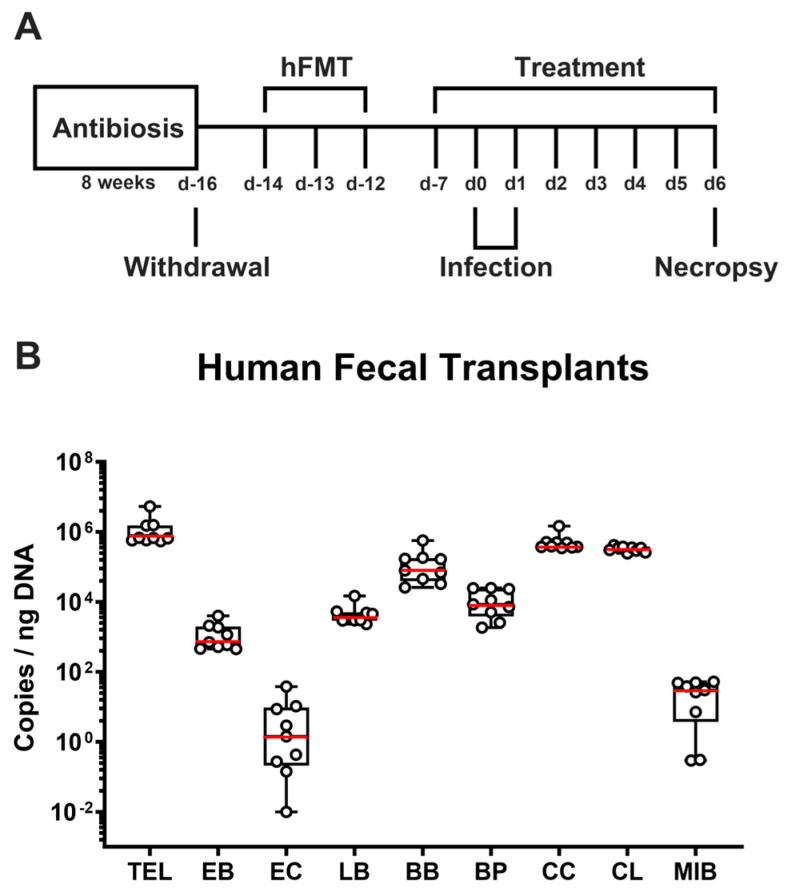
Timeline of experimental procedures and the microbiota composition of human fecal transplants. (**A**) Human microbiota-associated (hma) IL-10^−/−^ mice were generated by antibiotic gut microbial depletion (with ampicillin plus sulbactam) and subsequent triple human fecal microbiota transplantation (hFMT). Starting a week before oral *Campylobacter jejuni* strain 81–176 infection, hma mice were subjected to menthol treatment via the drinking water and followed up until necropsy on day (d) 6 post-infection. (**B**) Gut microbial communities were quantitated in human fecal transplants (n = 3 per experiment) by culture-independent (i.e., 16S rRNA-based molecular) analyses. Box plots (25th and 75th percentiles), whiskers (minimum and maximum values), and medians (red bar in boxes) are given (pooled from three independent experiments). TEL, total eubacterial loads; EB, enterobacteria; EC, enterococci; LB, lactobacilli; BB, bifidobacteria; BP, *Bacteroides/Prevotella* species; CC, *Clostridium coccoides* group; CL, *Clostridium leptum* (CL) group; MIB, Mouse Intestinal *Bacteroides*.

In the same unit of the Forschungseinrichtungen für Experimentelle Medizin (FEM, Charité-University Medicine Berlin), IL-10^−/−^ mice (of the C57BL/6 background) were bred under specific pathogen-free (SPF) and standardized conditions (i.e., 22–24 °C room temperature, 55 ± 15% humidity, and a 12 h light/12 dark cycle). In an experimental semi-barrier, mice were housed in cages with filter tops covering them and were allowed unrestricted access to autoclaved standard chow (food pellets: sniff R/M-H, V1534-300, Sniff, Soest, Germany). After placing 3-week-old littermate animals in sterile cages (two to four mice per cage), ampicillin and sulbactam (2 g/L and 1 g/L, respectively; Dr. Friedrich Eberth Arzneimittel, Ursensollen, Germany) were administered via the drinking water for eight weeks (ad libitum) as stated earlier [38,39]. The mice were handled under rigorous aseptic conditions to prevent microbial contamination and ensure successful microbiota depletion. Two days before human FMT (day-16; Figure 1A), the antibiotics were replaced with sterile tap water (ad libitum).

### 2.2. Human Fecal Microbiota Transplantation

Fresh fecal samples were voluntarily donated by healthy subjects, suspended in sterile phosphate-buffered saline (PBS; Thermo Fisher Scientific, Waltham, MA, USA), aliquoted, and preserved at −80 °C. Prior to the human FMT, fecal aliquots were thawed and pooled immediately. To establish hma mice, human fecal microbiota transplantation (hFMT) was performed over three consecutive days (days-14, -13, and -12; Figure 1A). Therefore, mice were orally subjected to 0.3 mL of the human fecal donor suspension via orogastric gavage, as outlined earlier [35].

### 2.3. Prophylactic Regimen

Commencing seven days prior to *C. jejuni* infection (day-7; Figure 1A), mice were treated with synthetic menthol (purchased from Sigma-Alderich, München, Germany). Menthol was administered to mice by autoclaved tap water (ad libitum). The final menthol concentration in the drinking solution was 500 mg/L, which meant that 100 mg of treatment dosage per kilogram of body weight was applied each day. Placebo-control mice received autoclaved tap water.

### 2.4. Campylobacter jejuni Infection

Bacterial cryo stocks of *C. jejuni* strain 81–176 were preserved at −80 °C. Following thawing, the bacteria were streaked out and cultured on selective karmali agar plates (obtained from Oxoid, Wesel, Germany) under microaerophilic conditions for 48 h at 37 °C, as previously reported [35]. The bacteria were then resuspended in sterile PBS (Thermo Fisher Scientific, Waltham, MA, USA), and the hma IL-10^−/−^ mice (female and male 3-month-old littermates) were infected with 10^9^ colony-forming units (CFU) of the enteropathogen on days 0 and 1 via orogastric gavage (Figure 1A).

### 2.5. Summary of Mouse Cohorts

Naive group (6 female/6 male): human FMT, non-treated, non-infected;

Placebo group (9 female/8 male): human FMT, non-treated, infected;

Menthol group (11 female/10 male): human FMT, verum-treated, infected.

### 2.6. Measurement of Gastrointestinal C. jejuni Loads

To assess gastrointestinal pathogen levels, the counts of live *C. jejuni* bacteria were monitored daily in fecal samples and additionally, upon necropsy in intraluminal gastrointestinal biopsies taken from the stomach, duodenum, ileum, and colon lumen that were homogenized in sterile PBS (Thermo Fisher Scientific, Waltham, MA, USA). *C. jejuni* quantification involved counting CFU after cultivating serial dilutions of intestinal samples on karmali agar for minimum 48 h at 37 °C under microaerophilic conditions as previously described in details [35]. The detection limit for viable pathogens was in the range of 100 CFU of *C. jejuni* bacteria per gram fecal material.

### 2.7. Fecal Microbiota Composition

For the culture-independent molecular analysis of the fecal microbiota composition, genomic DNA was isolated from the human fecal transplants and the colonic luminal murine samples first. In a nutshell, the Quant-iT PicoGreen reagent (Invitrogen, Paisley, UK) was used to quantify the DNA content, which was then adjusted to 1 ng of DNA per µL. Using species-, genera-, or group-specific 16S rRNA gene primers (Tib MolBiol, Berlin, Germany), the most prominent bacterial groups that were abundant in the intestinal microbiota of the hma mice were evaluated using quantitative real-time polymerase chain reaction (qRT-PCR), as previously described [40].

### 2.8. Clinical Conditions of Mice

We used a standardized cumulative clinical score on a daily basis to record the clinical status of mice before and after enteropathogen application as reported earlier [41] (Supplementary Table S1).

### 2.9. Sampling Procedures

Following *C. jejuni* infection, mice were sacrificed on day 6 p.i. by inhalation of carbon dioxide. Luminal gastrointestinal samples were obtained under sterile conditions from the stomach, duodenum, ileum, and colon, in addition to ex vivo biopsies from the mesenteric lymph nodes (MLN) and the distal colon, for subsequent microbiological, immunohistopathological, and immunological measurements. Cardiac blood was taken to generate serum samples.

### 2.10. Histopathology

Colon explants were promptly fixed in 5% formalin, embedded in paraffin, and 5-µm-sections were subjected to hematoxylin and eosin (H&E) staining. To evaluate the severity of histological alterations of the colonic mucosa, the tissue samples were assessed using light microscopy (100-times magnification), and the histopathological sequelae of infection were quantified based on an established scoring system [42] (Supplementary Table S2).

### 2.11. Quantitative In Situ Immunohistochemistry

As previously outlined in detail [43], quantitative in situ immunohistochemistry investigations were conducted on large intestinal explants that had been promptly subjected to tissue fixation in 5% formalin paraffin embedding. Subsequently, 5-µm-sections of paraffin-embedded tissues were stained with distinct primary antibodies (Supplementary Table S3). A blinded and independent researcher used light microscopy (400-times magnification) to enumerate the positively stained cells. For each sample, the mean count of specifically stained cells was determined across a minimum of six high-power fields (HPFs, 0.287 mm^2^, 400-times magnification).

### 2.12. Pro-Inflammatory Mediators

Terminal ileal and colonic explants (approximately 1 cm^2^ each) were longitudinally cut and the tissues cleansed in sterile PBS (Thermo Fisher Scientific, Waltham, MA, USA). Then, in addition to the intestinal samples, ex vivo biopsies derived from the MLN (3 nodes) were transferred to 24-flat-bottom well culture plates (Thermo Fisher Scientific, Waltham, MA, USA) containing 500 µL serum-free RPMI 1640 medium (Thermo Fisher Scientific, Waltham, MA, USA), penicillin (100 µg/mL; Biochrom, Berlin, Germany) and streptomycin (100 µg/mL; Biochrom, Berlin, Germany). Following an 18 h incubation period at 37 °C, culture supernatants were tested for monocyte chemoattractant protein-1 (MCP-1) by the Mouse Inflammation Cytometric Bead Assay (BD Biosciences, Heidelberg, Germany) in a BD FACSCanto II flow cytometer (BD Biosciences). Nitric oxide (NO) levels were determined by the Griess reaction [44].

### 2.13. Statistics

GraphPad Prism (version 9; San Diego, CA, USA) was used to compute medians and significance levels following the pooling of data from three independent experiments. The normal distribution of the data was evaluated using the Anderson-Darling test. Pairwise comparisons of regularly distributed and non-normally distributed data were performed using the Student’s *t*-test and the Mann–Whitney test, respectively. The one-way ANOVA with Tukey post hoc test (for regularly distributed data) and the Kruskal–Wallis test with Dunn’s post hoc test (for non-normally distributed data) were used for multiple comparisons. Significant two-sided probability (*p*) values were defined as <0.05. Significant outliers were determined by using the Grubb’s test (α = 0.001).

## 3. Results

### 3.1. Clinical Conditions over Time following C. jejuni Infection of hma IL-10^−/−^ Mice with Menthol Pretreatment

**Figure 2:**
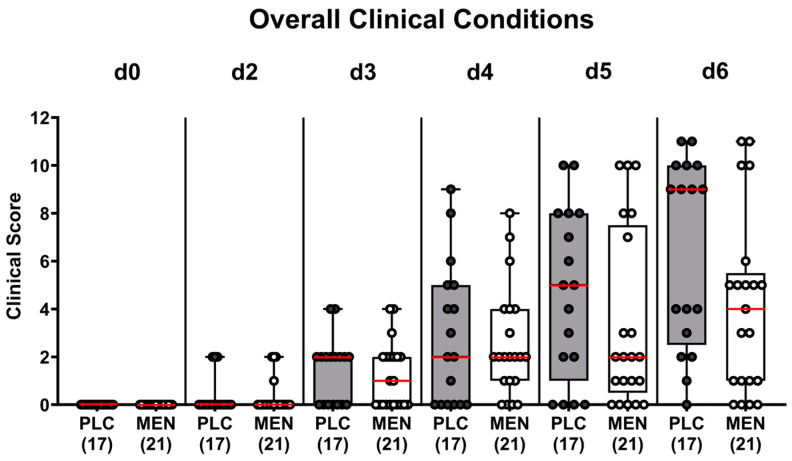
Clinical conditions over time following *C. jejuni* infection of hma IL-10^−/−^ mice with menthol pretreatment. Hma mice were orally pretreated with menthol (MEN, white bars) or placebo (PLC, grey bars) and infected with *C. jejuni* on day 0 (d0) and d1 by orogastric gavage. The clinical conditions of mice were surveyed daily until necropsy by using a clinical campylobacteriosis score recording wasting, diarrhea, and fecal blood (see methods). Box plots (25th and 75th percentiles), whiskers (minimum and maximum values), medians (red line in boxes), and numbers of analyzed mice (in parentheses) from three experiments are given.

First, we tested whether menthol pretreatment would improve the clinical conditions in hma IL10^−/−^ mice following *C. jejuni* infection. Our daily assessments revealed that mice from the menthol and placebo cohorts presented with comparable campylobacteriosis-related conditions at individual days p.i. (not significant (n.s.); Figure 2). This was also the case when focusing on clinical scores specifying wasting symptoms, diarrhea, and fecal flood (n.s.; Supplementary Figure S1). Notably, the standard deviations of the respective clinical scores were very high within both infected groups. Hence, menthol pretreatment did not alleviate campylobacteriosis symptoms.

### 3.2. Pathogen Numbers in Gastrointestinal Luminal Samples from C. jejuni Infected hma IL-10^−/−^ Mice with Menthol Pretreatment

**Figure 3:**
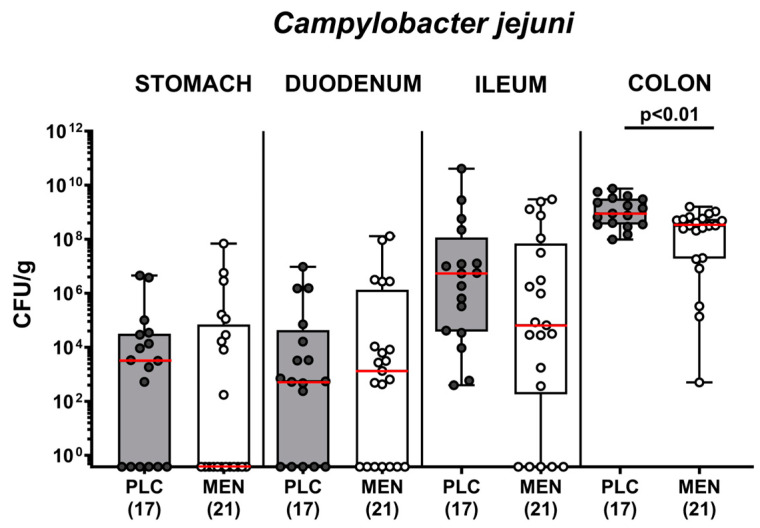
Pathogen numbers in gastrointestinal luminal samples from *C. jejuni* infected hma IL-10^−/−^ mice with menthol pretreatment. Hma mice were orally pretreated with menthol (MEN, white bars) or placebo (PLC, grey bars) and infected with *C. jejuni* on days 0 and 1 by orogastric gavage. The luminal *C. jejuni* numbers were determined in gastrointestinal samples taken on day 6 post-infection by culture. Box plots (25th and 75th percentiles), whiskers (minimum and maximum values), medians (red line in boxes), significance levels (*p* values) determined by the Mann–Whitney test, and numbers of analyzed mice (in parentheses) from three experiments are given. CFU, colony-forming units.

Next, we addressed whether oral menthol would interfere with intestinal *C. jejuni* colonization. Results from daily cultural analyses indicated comparably high *C. jejuni* numbers in fecal samples over time following infection of mice from both cohorts (n.s.; Supplementary Figure S2). On the day of necropsy (i.e., day 6 p.i.), luminal *C. jejuni* loads did not differ in the stomach, duodenum, and ileum of menthol and placebo-pretreated mice (n.s.), whereas less than one order of magnitude lower median pathogen numbers were detected in the colonic lumen of the former vs. the latter (*p <* 0.01; Figure 3). Hence, menthol pretreatment slightly but significantly lowered colonic *C. jejuni* numbers.

### 3.3. Inflammatory Changes in the Colon of C. jejuni Infected hma IL-10^−/−^ Mice with Menthol Pretreatment

**Figure 4:**
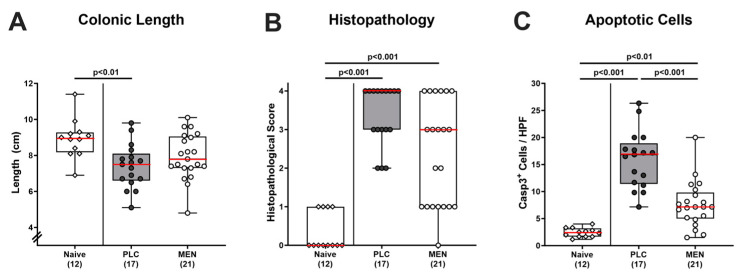
Inflammatory changes in the colon of *C. jejuni* infected hma IL-10^−/−^ mice with menthol pretreatment. Hma IL-10^−/−^ mice were orally pretreated with menthol (MEN, white bars) or placebo (PLC, grey bars) and infected with *C. jejuni* on days 0 and 1 by orogastric gavage. On day 6 post-infection, (**A**) the colonic lengths were measured. Furthermore, (**B**) the histopathological scores and (**C**) numbers of apoptotic epithelial cells positive for cleaved caspase-3 (Casp3^+^) were determined in colonic paraffin sections. Naive mice (non-infected without pretreatment) served as negative controls. Box plots (25th and 75th percentiles), whiskers (minimum and maximum values), medians (red line in boxes), significance levels (*p* values) determined by the one-way ANOVA test with Tukey post hoc test (**A**,**C**) and Kruskal–Wallis test with Dunn’s post hoc test (**B**), and numbers of analyzed mice (in parentheses) from three experiments are given. HPF, high power field.

Further, we tested whether menthol pretreatment could mitigate *C. jejuni*-induced macroscopic and microscopic inflammatory changes. As a macroscopic inflammatory parameter, we recorded the absolute lengths of the large intestines upon necropsy. On day 6 p.i., we found shorter colons in mice from the placebo (*p <* 0.01) as opposed to the menthol cohort (n.s.) when compared to naive (i.e., non-treated and non-infected) control animals (Figure 4A), indicative of mitigated macroscopic inflammatory signs of *C. jejuni*-infection. When addressing the severity of microscopic inflammatory sequelae in the colon, we revealed comparable histopathological scores in mice from both treatment cohorts but observed a trend towards lower values in the menthol group as compared to the placebo group that did not reach statistical significance due to high standard deviations in the former (Figure 4B). We further investigated the extent of *C. jejuni*-induced intestinal apoptosis and found increased numbers of apoptotic cells in colonic epithelia in both infected groups (*p <* 0.01–0.001), but considerably fewer apoptotic cell counts in menthol as compared to placebo-treated mice on day 6 p.i. (*p <* 0.001; Figure 4C). Hence, menthol pretreatment alleviated both macroscopic and microscopic complications of the *C. jejuni* infection.

### 3.4. Colonic Immune Cells in C. jejuni Infected hma IL-10^−/−^ Mice with Menthol Pretreatment

**Figure 5:**
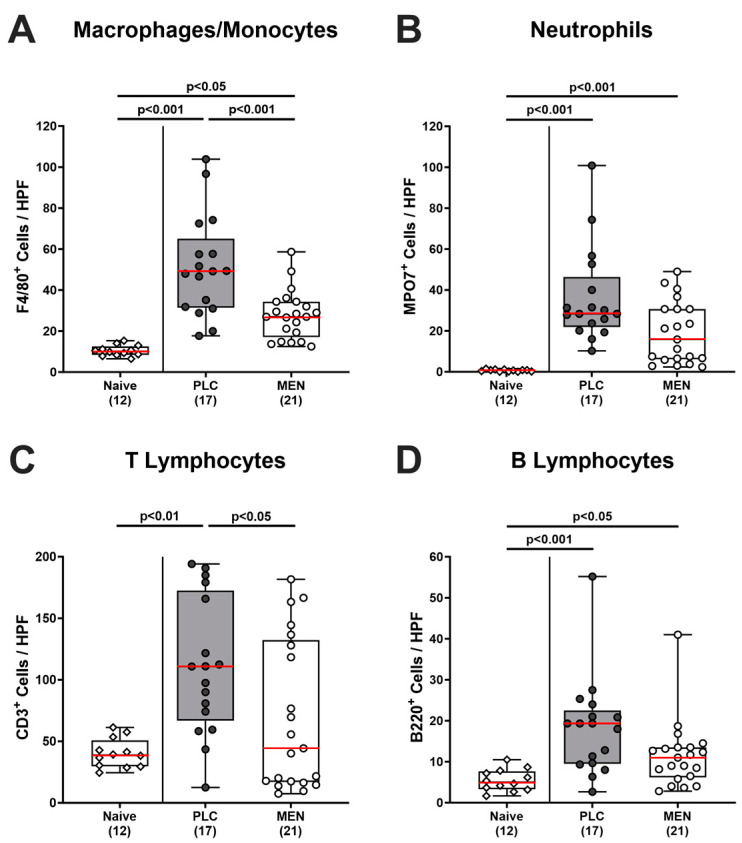
Colonic immune cells in *C. jejuni* infected hma IL-10^−/−^ mice with menthol pretreatment. Hma IL-10^−/−^ mice were orally pretreated with menthol (MEN, white bars) or placebo (PLC, grey bars) and infected with *C. jejuni* on days 0 and 1 by orogastric gavage. On day 6 post-infection, numbers of (**A**) macrophages and monocytes (F4/80^+^), (**B**) neutrophils (MPO7^+^), (**C**) T lymphocytes (CD3^+^), and (**D**) B lymphocytes (B220^+^) were determined in the colonic lamina propria (immunohistochemically stained paraffin sections). Naive mice (non-infected without pretreatment) served as negative controls. Box plots (25th and 75th percentiles), whiskers (minimum and maximum values), medians (red line in boxes), significance levels (*p* values) determined by the one-way ANOVA test with Tukey post hoc test (**A**) and Kruskal–Wallis test with Dunn’s post hoc test (**B**–**D**), and numbers of analyzed mice (in parentheses) from three experiments are given. HPF, high power field.

Potential menthol-dependent immune cell responses upon *C. jejuni* infection were investigated by in situ immunohistochemical analyses in colonic paraffin sections. Results demonstrated that *C. jejuni* infection was accompanied by increases in innate immune cell populations such as F4/80^+^ macrophages and monocytes (*p <* 0.05–0.001; Figure 5A) and MPO7^+^ neutrophilic granulocytes (*p <* 0.001; Figure 5B) in the colonic mucosa and lamina propria. The increase in the former was, however, less pronounced in menthol as compared to placebo-pretreated mice (*p <* 0.001; Figure 5A). In the case of the neutrophils, at least a trend towards fewer cell counts could be observed upon menthol challenge on day 6 p.i. (n.s. vs. placebo; Figure 5B). When assessing *C. jejuni*-induced adaptive immune cell responses, we detected increased colonic numbers of CD3^+^ T lymphocytes in placebo (*p <* 0.01 vs. naive) but not in menthol-pretreated mice (*p <* 0.05 vs. placebo; n.s. vs. naive) on day 6 p.i. (Figure 5C). Like neutrophils, *C. jejuni*-infected mice displayed elevated B220^+^ B lymphocyte numbers in their colonic mucosa and lamina propria (*p <* 0.05–0.001; Figure 5D), whereas a trend towards lower counts was detected in menthol-treated mice as compared to placebo-treated mice on day 6 p.i. (n.s.; Figure 5D). Hence, menthol pretreatment resulted in attenuated recruitment of macrophages/monocytes and of T lymphocytes into the colon upon *C. jejuni* infection.

### 3.5. Intestinal Proinflammatory Mediator Secretion in C. jejuni Infected hma IL-10^−/−^ Mice with Menthol Pretreatment

**Figure 6:**
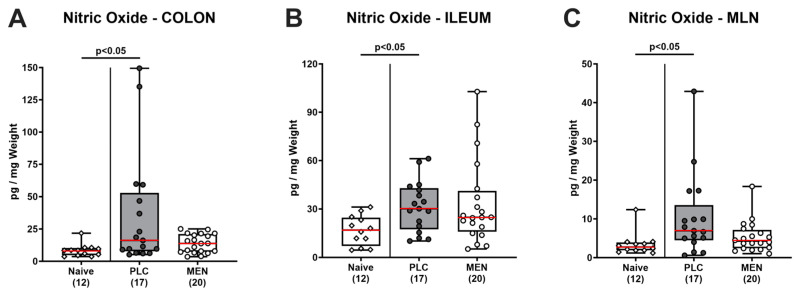
Intestinal nitric oxide secretion in *C. jejuni* infected hma IL-10^−/−^ mice with menthol pretreatment. Hma IL-10^−/−^ mice were orally pretreated with menthol (MEN, white bars) or placebo (PLC, grey bars) and infected with *C. jejuni* on days 0 and 1 by orogastric gavage. On day 6 post-infection, nitric oxide concentrations were measured in ex vivo biopsies sampled from the (**A**) colon, (**B**) ileum, and (**C**) mesenteric lymph nodes (MLN). Naive mice (non-infected without pretreatment) served as negative controls. Box plots (25th and 75th percentiles), whiskers (minimum and maximum values), medians (red line in boxes), significance levels (*p* values) determined by the Kruskal–Wallis test with Dunn’s post hoc test, and numbers of analyzed mice (in parentheses) from three experiments are given. The Grubb’s test was used to identify definite outliers.

Next, we determined NO concentrations in distinct intestinal organs. Whereas enhanced NO secretion could be detected in ex vivo biopsies derived from the colon, ileum, and MLN of placebo control animals on day 6 p.i. (*p <* 0.005 vs. naive), menthol-pretreated mice displayed basal values (n.s. vs. naive; Figure 6), indicative of diminished *C. jejuni*-induced oxidative stress in respective intestinal compartments. Furthermore, *C. jejuni* infection resulted in increased colonic secretion of the pro-inflammatory chemokine MCP-1 in mice from the placebo group (*p <* 0.05 vs. naive), but not in the menthol cohort (n.s. vs. naive; Supplementary Figure S3). Hence, menthol pretreatment resulted in attenuated *C. jejuni*-induced intestinal secretion of pro-inflammatory mediators, thereby reducing oxidative stress in the intestinal organs.

### 3.6. Fecal Microbiota during C. jejuni Infection of hma IL-10^−/−^ Mice with Menthol Pretreatment

**Figure 7:**
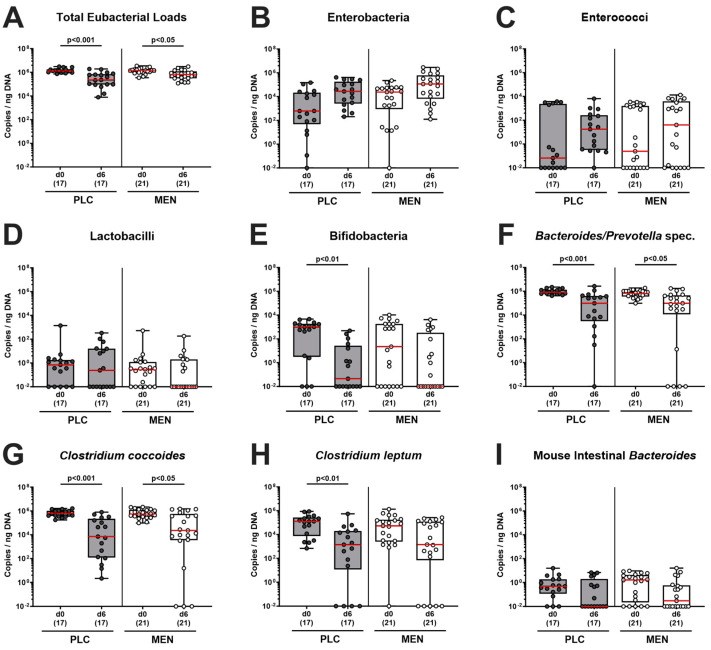
Fecal microbiota during *C. jejuni* infection of hma IL-10^−/−^ mice with menthol pretreatment. Immediately before (i.e., day (d)0) and on d6 post *C. jejuni* infection, the fecal microbiota compositions were assessed in hma IL-10^−/−^ mice with menthol (MEN; white bars) or placebo (PLC; grey bars) prophylaxis by culture-independent methods. The (**A**) total eubacterial loads, (**B**) enterobacteria, (**C**) enterococci, (**D**) lactobacilli, (**E**) bifidobacteria, (**F**) *Bacteroides/Prevotella* species, (**G**) *Clostridium coccoides* and (**H**) *Clostridium leptum* groups, and (**I**) Mouse Intestinal *Bacteroides* are expressed as copies per ng DNA. Box plots (25th and 75th percentiles), whiskers (minimum and maximum values), medians (red line in boxes), significance levels (*p* values) determined by the one-way ANOVA test with Tukey post hoc test (**A**) and the Kruskal–Wallis test with Dunn’s post hoc test (**B**–**I**), and numbers of analyzed mice (in parentheses) from three experiments are given.

Furthermore, we tested for fecal microbiota changes during *C. jejuni* infection in menthol-treated mice. Our quantitative culture-independent analyses revealed comparable fecal microbiota compositions in both cohorts on day 0, and hence, immediately before oral *C. jejuni* challenge of mice (n.s. menthol vs. placebo; Figure 7). In the course of *C. jejuni* infection, total eubacterial loads and gene numbers of *Bacteroides/Prevotella* species and of the *Clostridium coccoides* group decreased irrespective of the pretreatment (*p <* 0.05–0.001, day 6 vs. day 0; Figure 7A,F,G). As opposed to menthol-pretreated mice, the gene copies of bifidobacteria (*p <* 0.01; Figure 7E) and of the *Clostridium leptum* group (*p <* 0.01; Figure 7H) were lower in fecal samples taken from placebo counterparts on day 6 p.i. if compared to day 0. Hence, oral menthol pretreatment had (if at all) only minor effects on the human fecal gut microbiota composition during *C. jejuni* infection of hma IL-10^−/−^ mice.

## 4. Discussion

In our present preclinical placebo-controlled intervention study, we tested the disease-mitigating, anti-pathogenic, anti-inflammatory, anti-oxidant and anti-apoptotic effects of oral menthol in acute experimental campylobacteriosis employing hma IL-10^−/−^ mice. At first, it was rather disappointing that menthol pretreatment did not significantly improve overt *C. jejuni*-induced clinical signs such as wasting symptoms and bloody diarrhea (Figure 2). One needs to take into consideration, however, that the severity of the observed symptoms ranged from almost no observable clinical signs at all to full-blown disease in both cohorts. In our previous in vivo study employing a different murine campylobacteriosis model, we found that oral menthol of the same concentration therapeutically given to secondary abiotic IL-10^−/−^ mice from day 2 until day 6 p.i. resulted in an improved clinical outcome [45].

In our actual trial, oral menthol pretreatment could prevent inflammation-induced colonic shrinkage, whereas placebo-treated mice displayed shorter colons upon *C. jejuni* infection if compared to untreated and non-infected controls (Figure 4A). The anti-inflammatory effects of exogenous menthol were also evident on the microscopic level, given that treated animals displayed diminished pathogen-induced colonic epithelial apoptosis (Figure 4C). In support, previous in vivo studies reported potent anti-apoptotic effects upon menthol treatment, given decreases in pro-apoptotic molecules such as cleaved caspase-3 and Bax protein and/or increases in anti-apoptotic molecules including Bcl-2 and HSP-70 [32,33,46,47]. The dampened apoptotic responses upon *C. jejuni* infection were accompanied by lower numbers of macrophages and monocytes in the colonic mucosa in menthol-pretreated mice on day 6 p.i. (Figure 5A). In support, menthol application could decrease infiltration of infected brain tissues with respective innate immune cell subsets during brain ischemia [48]. Furthermore, *C. jejuni*-induced increases in colonic T lymphocytes could be reversed to basal values in menthol-treated mice (Figure 5C). In support, a recent in vitro study revealed that menthol inhibited lymphocytic proliferation in a dose-dependent manner and reduced the numbers of T cells expressing pro-inflammatory cytokines [49]. Moreover, in our study, oral menthol could effectively reduce pathogen-induced oxidative stress in different intestinal compartments, as shown by NO concentrations measured in the colon, ileum, and MLN of treated mice on day 6 p.i. that did not differ from those obtained from naive mice (Figure 6). In parallel, enhanced colonic secretion of the pro-inflammatory chemokine MCP-1 secretion could be assessed in the infected placebo as opposed to the verum cohort upon necropsy (Supplementary Figure S3).

The potent anti-oxidant and anti-inflammatory properties of exogenous menthol were shown in vitro to inhibit NO and pro-inflammatory mediator secretion in LPS-stimulated monocytes [50,51]. Furthermore, in vivo studies revealed that menthol mitigated acute intestinal morbidities. Intraperitoneal injection of synthetic menthol before colitis induction, for instance, could exert potent anti-colitogenic effects in rats [52], which also held true for an improved clinical outcome and attenuated macroscopic and microscopic disease due to diminished oxidative stress and inflammation in rats suffering from acute colitis [53].

When addressing the anti-pathogenic effects of the compounds, we found less than one order of magnitude lower *C. jejuni* numbers in the colon lumen of menthol as compared to placebo treated mice on day 6 p.i. (Figure 3). It is, however, questionable whether the (statistically significant) slightly lower pathogen burden might explain the observed biological effects. In our previous study, we did not observe significant differences in colonic *C. jejuni* counts in menthol- vs. placebo-treated mice with acute campylobacteriosis. In that study, however, we applied a therapeutic regimen to secondary abiotic IL-10^−/−^ mice from day 2 until day 6 p.i. only [45]. Furthermore, the menthol concentration in the drinking solutions given in our previous and actual studies (i.e., 500 mg/L) was far below the minimum inhibitory concentration (MIC) of the compound measured against the applied *C. jejuni* 81–176 strain (16,000 mg/L). Anyhow, it is much more likely that the mitigated enterocolitis following menthol pretreatment of *C. jejuni*-infected mice was due to immune-modulatory, including anti-inflammatory, anti-oxidant, and anti-apoptotic effects, as, in support, had been reported in a murine gastritis model earlier [32].

Furthermore, we surveyed the impact of menthol pretreatment on influencing the (human) commensal gut microbiota composition following human FMT but found comparable fecal microbiota conditions on day 0 (i.e., immediately before *C. jejuni* infection) (Figure 7). This result points towards comparably effective engraftments of fecal human transplants and speaks against the significant microbiota-shifting effects of exogenous menthol that had already been applied for one week by that time point. In addition, loads of human bacterial communities were comparable in fecal samples derived at the end of the experiment on day 6 p.i. (Figure 7). When focusing on microbiota shifts during *C. jejuni* infection within respective treatment cohorts (i.e., changes between day 0 and day 6 p.i.), we found statistically significant decreases in fecal gene numbers of potentially probiotic bifidobacteria and *Clostridium leptum* in the placebo, but only a trend towards lower counts of respective phyla in the menthol cohort (n.s.; Figure 7). Given these rather marginal differences, one should not overemphasize these observations.

Menthol is generally considered a safe compound by the United States Food and Drug Administration and the Flavoring Extract Manufacturer’s Association, and only very rarely have intoxications resulting in dizziness, agitation, hallucination, convulsion, and coma been reported [54,55]. A medical integrative international organization (Natural Standards) has indicated a dose of up to 1 g per kg of body weight as fatal [54], whereas in our trial, mice received one tenth.

In summary, our actual preclinical trial provides the first evidence that oral pretreatment of *C. jejuni*-infected IL-10^−/−^ mice harboring a commensal human gut microbiota mitigates acute enterocolitis due to its immune-modulatory, including anti-inflammatory, anti-oxidant, and anti-apoptotic modes of action.

## 5. Conclusions

Menthol constitutes a promising antibiotic-independent option to fight acute campylobacteriosis, thereby decreasing the risk for the development of post-infectious autoimmune morbidities given that the severity of the enteritis is associated with the risk of complications including Guillain–Barré syndrome, reactive arthritis, and chronic intestinal inflammatory morbidities later on. Furthermore, menthol might be a useful measure to prevent *C. jejuni* infection in individuals with an increased risk of exposure to the enteropathogen. Future research should focus on developing antibiotic-independent methods for the prevention and/or treatment of food-borne illnesses brought on by enteropathogens besides *Campylobacter* species.

## Data Availability

The data presented in this study are available on request from the corresponding author.

## Supplementary Materials

The following supporting information can be downloaded at: https://www.mdpi.com/article/10.3390/biom14030290/s1, Table S1: Clinical scores; Table S2: Histopathological scores; Table S3: Primary antibodies for in situ immunohistochemical analyses; Figure S1: Clinical signs of campylobacteriosis over time following *C. jejuni* infection of hma IL-10^−/−^ mice with menthol pre-treatment. Figure S2: Pathogen numbers in fecal samples taken following *C. jejuni* infection of hma IL-10^−/−^ mice with menthol pre-treatment. Figure S3: Colonic MCP-1 secretion in *C. jejuni* infected hma IL-10^−/−^ mice with menthol pretreatment.
