# Supplementary material for: Menthol Pretreatment Alleviates Campylobacter jejuni-Induced Enterocolitis in Human Gut Microbiota-Associated IL-10−/− Mice
Source: Biomolecules. 2024 Feb 29;14(3):290. doi: 10.3390/biom14030290 (PMC10968592; doi:10.3390/biom14030290)
Supplement: Supplementary file 1 [file biomolecules-14-00290-s001.zip › biomolecules-2863142-supplementary.pdf]

biomolecules

MDPI

Menthol pretreatment alleviates *Campylobacter jejuni* induced enterocolitis in human gut microbiota-associated IL-10$^{-/-}$ mice

Markus M. Heimesaat[1#], Luis Q. Langfeld[1], Niklas Schabbel[1], Nizar W. Shayya[1], Soraya Mousavi[1], Stefan Bereswill[1]

[1] Gastrointestinal Microbiology Research Group, Institute of Microbiology, Infectious Diseases and Immunology, Charité - Universitätsmedizin Berlin, corporate member of Freie Universität Berlin, Humboldt-Universität zu Berlin, and Berlin Institute of Health, Berlin, Germany

Supplementary Table 1. Clinical scores (maximum 12 points).

Clinical aspect	Scores
**Wasting symptoms**	0: normal<br>1: ruffled fur<br>2: less locomotion<br>3: isolation<br>4: severely compromised locomotion, pre-final aspect
**Stool consistency**	0: formed feces<br>2: pasty feces<br>4: liquid feces
**Fecal blood**	0: no blood<br>2: microscopic detection of blood by the Guajac method using Haemoccult, Beckman Coulter/PCD, Germany<br>4: macroscopic blood visible

Supplementary Table 2: Histopathological scores (maximum 4 points).

**Score 0**	Normal epithelium without inflammatory cell infiltrates.
**Score 1**	Minimal inflammatory cell infiltrates in the mucosa with intact epithelium.
**Score 2**	Mild inflammatory cell infiltrates in the mucosa and submucosa with mild hyperplasia and mild goblet cell loss.
**Score 3**	Moderate inflammatory cell infiltrates in the mucosa with moderate goblet cell loss.
**Score 4**	Marked inflammatory cell infiltration into the mucosa and submucosa with marked goblet cell loss, multiple crypt abscesses, and crypt loss.

Supplementary Table 3: Primary antibodies for *in situ* immunohistochemical analyses

Cells	Primary antibody
Apoptotic epithelial cells	cleaved caspase-3 (Asp175, Cell Signaling, Beverly, MA, USA, 1:200)
Macrophages/monocytes	F4/80 (no. 14-4801, clone BM8, eBioscience, San Diego, CA, USA, 1:50
Neutrophils	MPO7 (No. A0398, Dako, Glostrup, Denmark, 1:500)
T lymphocytes	CD3 (no. N1580, Dako, 1:10)
B lymphocytes	B220 (no. 14-0452-81, eBioscience, San Diego, CA, USA;1:200).

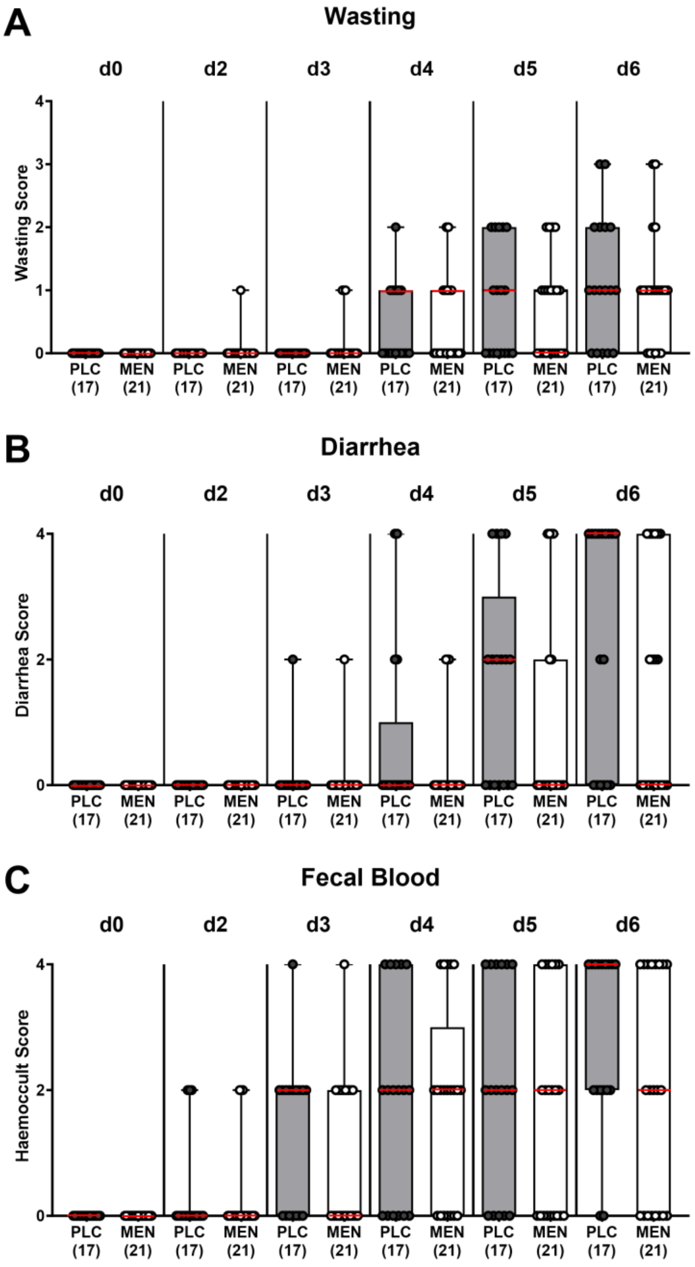

Supplementary Figure S1. Clinical signs of campylobacteriosis over time following *C. jejuni* infection of hma IL-10$^{-/-}$ mice with menthol pre-treatment. Hma IL-10$^{-/-}$ mice were orally pretreated with menthol (MEN, white bars) or placebo (PLC, grey bars) and infected with *C. jejuni* on day 0 (d0) and d1 by gavage. The clinical signs of campylobacteriosis such as **(A)** wasting symptoms, **(B)** diarrhea, and **(C)** fecal blood were recorded with individual scores (see methods). Box plots (25th and 75th percentiles), whiskers (minimum and maximum values), medians (red bar in boxes), and numbers of analyzed mice (in parentheses) from three experiments are given.

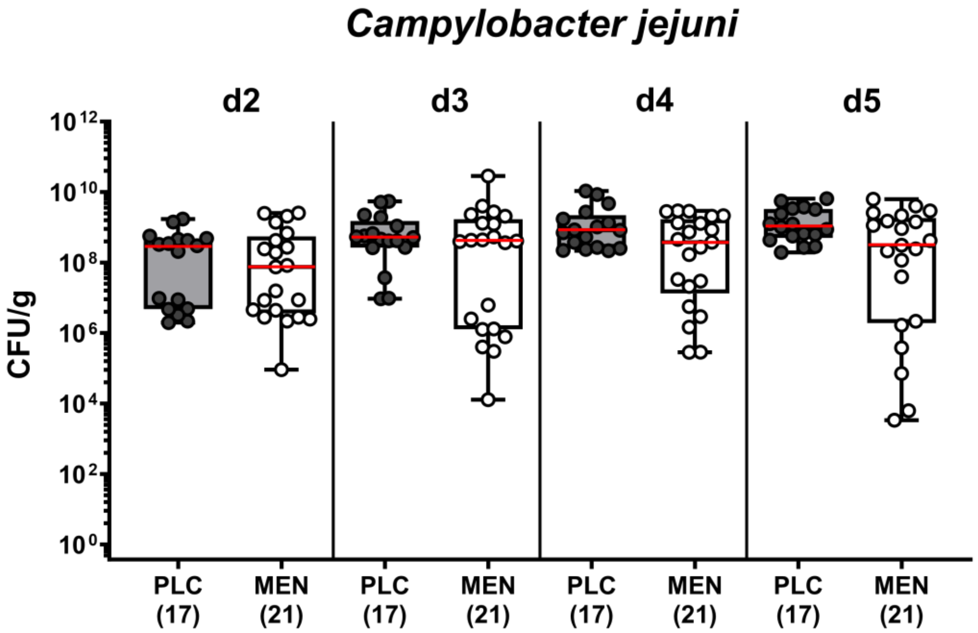

Supplementary Figure S2. Pathogen numbers in fecal samples taken following *C. jejuni* infection of hma IL-10$^{-/-}$ mice with menthol pre-treatment. Hma IL-10$^{-/-}$ mice were orally pretreated with menthol (MEN, white bars) or placebo (PLC, grey bars) and infected with *C. jejuni* on day 0 (d0) and d1 by gavage. The fecal C. jejuni numbers were determined at defined time points post-infection by culture. Box plots (25th and 75th percentiles), whiskers (minimum and maximum values), medians (red bar in boxes), and numbers of analyzed mice (in parentheses) from three experiments are given. CFU, colony-forming units.

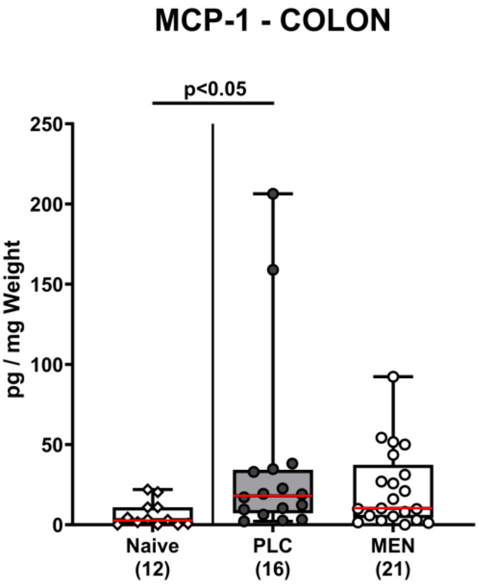

Supplementary Figure S3. Colonic MCP-1 secretion in *C. jejuni* infected hma IL-10$^{-/-}$ mice with menthol pretreatment. Hma IL-10$^{-/-}$ mice were orally pretreated with menthol (MEN, white bars) or placebo (PLC, grey bars) and infected with *C. jejuni* on days 0 and 1 by gavage. On day 6 post-infection, MCP-1 concentrations were measured in *ex vivo* biopsies sampled from the colon. Naive mice (non-infected without pretreatment) served as negative controls. Box plots (25th and 75th percentiles), whiskers (minimum and maximum values), medians (red bar in boxes), significance levels (p values) determined by the Kruskal-Wallis test with Dunn's post hoc test, and numbers of analyzed mice (in parentheses) from three experiments are given. The Grubb's test was used to identify definite outliers.
